# Medical News

**Published:** 1851-10

**Authors:** 


### THE MEDICAL EXAMINER.

PHILADELPHIA, OCTOBER, 1851.

### MEDICAL NEWS.

British Provincial Medical and Surgical Association.—The Nineteenth Anniversary of this Association was held at Brighton, Wednesday, 13th of August last; Dr. Jenks in the chair.

Contemplated Organization of the Profession in Kentucky. A representation of the Physicians of Kentucky, is to assemble in convention at Frankfort in that state, on the 1st of October, with a view to the organization of a State Medical Society.

Dr. Henderson and the University of Edinburgh.— The Faculty of Medicine of the University of Edinburgh, is endeavoring to eject Dr. Henderson, Professor of General Pathology, from his chair, on the ground of his being an avowed homœopathist. The Senatus Academicus, consisting of the professors of all the faculties, refuses to sustain the Medical Faculty, and there the matter rests for the present, "with the possibility that the Medical Faculty may appeal directly to the Town Council."

Lying-in Hospital of the Faculty, at Paris.—A change has lately been introduced at this hospital in the arrangements regarding new-born children. It had been observed that a great many lying-in women being assembled, *with their infants,* in one ward, the cries of the latter interfered materially with the rest of all the patients; it therefore occurred to the head midwife of the establishment to place all the infants away from their mothers, into a separate ward, where the little creatures are kept very cleanly, and have at once whatever medical assistance they may require.

There can be no doubt that rest is extremely advantageous to lying-in women, and that the atmosphere of a ward with so many mothers and infants must be prejudicial to the latter; but it may be questioned whether the frequent conveyance of sucklings to their mothers, both night and day, from one ward to another, is a circumstance free from risk to the infants. It seems, after all, much simpler to multiply the wards, and have them smaller, than part mother and child in the manner described.—*London Lancet, Sept. 6th.*

Cutaneous and Muscular Galvanism in Paris.—Dr. Duchenne, (of Boulogne,) supported by most of the leading physicians of Paris, has lately introduced a peculiar mode of therapeutically using electricity. He calls it localized electrization, and it is principally to the skin and muscles that he applies, either by contact or induction, the currents produced by his peculiarly constructed instruments. Among the affections under the control of Dr. Duchenne's electrization, are sciatica, muscular rheumatism, hyperæsthesia, anæsthesia, &c., &c. He likewise stimulates directly the nervous expansions of internal organs, in cases of paralysis of such canals: as the rectum and its muscles; the bladder (for which a staff ending in a knob is passed into the rectum, and a catheter isolated with India-rubber, except where it is to come into contact with the muscular fibres, is introduced into the bladder;) the uterus (for which cavity a peculiar instrument is used;) the pharynx and œsophagus; the larynx; and by the instrumentality of the pneumo-gastric nerve, the stomach, liver, lungs, and heart.

The inventor uses his peculiar method for the excitation of the organs of sense, and the genito-urinary apparatus. One of the most striking innovations of Dr. Duchenne is the clever manner in which he succeeds in exciting single muscles, this faculty being of vast importance in the treatment of partial paralysis. We had ourselves an opportunity of seeing the manner in which Dr. Duchenne causes the contraction of any given muscle, at a demonstration lately given by him in London, at the house of Dr. H. Bennett, in the presence of several distinguished members of the profession. The experiments were made upon the face and arm of a groom, who volunteered his services; and by a peculiar mode of adapting the electricity by induction, and the pole with which the muscles were singled out, the inventor was able to foretel what kind of contraction of the arm or grimace in the face would be made. Our readers will find in the *Archives de Médecine*, vol. xxii. p. 263, and the February and March numbers, 1851, satisfactory details on this new method, of which our description will only give a faint idea.

We should not omit to state that Dr. Duchenne closes a paper (*Archives*, May, 1851) on the subject by these words: "As it will be useful to create a word which should excatly point out electricity by induction, as well as its application, may it not be allowable to use the name of the philosopher who has discovered this kind of electricity? Thus, in the same way as 'Galvani' has given his name to the electricity by contact, so can we likewise give to the electricity by induction the name of Faraday. This electricity would then be called 'Faradism,' and its application 'Faradization.' Such names would establish a clear distinction between the electricity by contact and that by induction, whilst they, at the same time, render due honor to a philosopher to whom medical science owes a discovery far more valuable in a therapeutical point of view than that of Galvani."—*Ibid, Sept.* 8*th.*

Clairovoyance in the seventeenth century.—"*April* 11*th.* 1681.—Honoured Sir,—I did receive your last letter, dated the 9th of this month, with much grief, haveing an account of your painfull feaver: I pray God it will not vex your body too much; and if by chance it should vex you longer, there is here a man that can cure it with sympathetical power; if you please to send me down the pearinghs of the nailes of both your hands and your foots, and three locks of hair of the top of your crown, I hope, with the grace of God, it will cure you. As for the compositions of them two masters, in my judgement, though weak, I like better Baptist's work than Pedro's, because Baptist work masterly, as you shall perceive, betwixt their bases. All Baptist's bases are singable where many of Pedro's are not so. Herewith my humble respects remaine, Honoured Sir, your most faithfull Servt., Cesare Morelli." —*Diary of Sam. Pepys, Third Edition, by Lord Braybrooke*, 1851, vol. v. p. 308. [He, (Cesare Morelli) appears to have been a music-master. Many of his compositions are preserved in the Pepysian Library.]

The Disease that has now for several years infested and destroyed the potato-crops, appears to be wearing out. Its ravages this season are evidently comparatively slight, except in Meath and Louth, in the province of Leinster. In some localities, where its traces have been detected, it presents so few of its old destructive features, as to be regarded with but little alarm. A month ago, however, there was dire apprehension as to the extent and severity of its ravages. Turnips will be, it is said, a partial failure, owing to the ravages of the diamond black moth; in some instances, this new plague has manifested itself to such an extent, that, in four or five days, the most luxuriant crop has been stripped of every green leaf, the mere stumps of the leaf-stalks remaining, where, a few days previously, the ground was covered with a healthy and vigorous vegetation.—*Lond. Med. Times.*

The Medical Officers of the Parisian Hospitals.—A Bill has been introduced before the French Assembly for the regulation of hospitals, in which was a clause recommending that the physicians and surgeons should be appointed by the Administration. M. Schælcher proposed that the candidates should undergo a public examination, (the Concours,) and that the most successful should be selected. The Amendment was rejected by 391 votes to 204, and the entire Bill was definitively adopted. This new regulation, we presume, does away with the principle of Concours, as far, at least, as hospital appointments are concerned; nor do we regret it, as students fresh from the schools, and mere book-men, under its influence, were selectable in preference to hard-working, practical men. Patients in hospitals require the services of men who can correctly diagnose the diseases, and apply the mode of treatment most appropriate for their cure; not those of men who are able to describe the minute anatomy, physiology, and chemistry of the human frame, but who are often worse than useless in matters of actual practice.—*Ibid.*

Extraordinary Accident.—A man was lately admitted into the Portsmouth, Portsea, and Gosport Hospital, under the following singular circumstances:—He was trying to extract a cork from a large stone beer-bottle with his teeth, when it was suddenly driven into his gullet by the force of the carbonic acid which had been generated in the bottle. Medical assistance was immediately obtained, but unavailingly, and the man was taken to the hospital, where œsophagotomy was at once practised, and the cork, which measured about three inches and a half in circumference was extracted.

Apparatus for Transfusion.—The London Medical Times, for the 6th of September, in its notices of objects connected with medicine and surgery to be seen at the Great Exhibition, gives the following account of a very simple and ingenious apparatus for transfusion, invented by Mr. Whitehead. Its consists of a graduated glass funnel for receiving the blood, connected by a piece of flexible tube, with a glass tube about eighteen inches long, at the lower end of which is another piece of flexible tube several inches in length, to which the pipe is attached. On the lower piece of flexible tube is fixed a metallic ring, with a screw which compresses or opens the tube, and prevents or allows the flow of the blood. The pipe introduced into the vein is of the ordinary shape, and is attached to a metal plate to be bound around the arm of the patient by a piece of ribbon or tape. This apparatus is much simplified by the absence of a syringe, the force by which the blood is introduced being that of a column of blood nearly three feet in height.

Prussian Despotism has just, at Breslau, deprived a physician, Dr. Borchard, of the right to practice his art—has withdrawn from him his medical license, because, in a speech delivered in the year 1848, he is said to have used treasonable language. Being tried for this offence at the time, he was condemned by a law tribunal to twelve year's imprisonment. On an appeal to another Court, this sentence was mitigated to three; which a third and final court of appeal has reduced to two. The Government, however, not finding this punishment sufficient, has, in order to increase it even beyond the rigor of the sentence, deprived the doctor for ever of his means of subsistence. The official document proclaiming this monstrous act of injustice and cruelty has been published in all official formality, in the *Neue Ober Zeitung* under the date of the 28th inst.

Obituary.—M. Teisseir, President of the Medical Society of the department of Aube, Director and Professor of the Obstetrical School of Troyes, died a short time ago, in consequence of a dissection wound made whilst lecturing on anatomy.—Dr. Vasse, Professor of Surgery at Bonn, died lately at Marburg, at the age of 73.—We mentioned at the beginning of this year, the death of Naegele, of Heidelberg. His son, likewise Professor at the University of Heidelberg, has just died, at the age of forty.—*London Lancet.*

